# Bacterial communities associated with honeybee food stores are correlated with land use

**DOI:** 10.1002/ece3.3999

**Published:** 2018-04-16

**Authors:** Philip Donkersley, Glenn Rhodes, Roger W. Pickup, Kevin C. Jones, Kenneth Wilson

**Affiliations:** ^1^ Lancaster Environment Centre Lancaster University Lancaster UK; ^2^ Lake Ecosystems Group Centre for Ecology and Hydrology Lancaster UK; ^3^ Division of Biomedical and Life Sciences Lancaster University Lancaster UK

**Keywords:** 16S rRNA, bacterial community, DGGE, honeybees, Illumina MiSeq, land use

## Abstract

Microbial communities, associated with almost all metazoans, can be inherited from the environment. Although the honeybee (*Apis mellifera* L.) gut microbiome is well documented, studies of the gut focus on just a small component of the bee microbiome. Other key areas such as the comb, propolis, honey, and stored pollen (bee bread) are poorly understood. Furthermore, little is known about the relationship between the pollinator microbiome and its environment. Here we present a study of the bee bread microbiome and its relationship with land use. We estimated bacterial community composition using both Illumina MiSeq DNA sequencing and denaturing gradient gel electrophoresis (DGGE). Illumina was used to gain a deeper understanding of precise species diversity across samples. DGGE was used on a larger number of samples where the costs of MiSeq had become prohibitive and therefore allowed us to study a greater number of bee breads across broader geographical axes. The former demonstrates bee bread comprises, on average, 13 distinct bacterial phyla; *Bacteroidetes*,* Firmicutes*,* Alpha‐proteobacteria*,* Beta‐proteobacteria,* and *Gamma‐proteobacteria* were the five most abundant. The most common genera were *Pseudomonas*,* Arsenophonus*,* Lactobacillus*,* Erwinia,* and *Acinetobacter*. DGGE data show bacterial community composition and diversity varied spatially and temporally both within and between hives. Land use data were obtained from the 2007 Countryside Survey. Certain habitats, such as improved grasslands, are associated with low diversity bee breads, meaning that these environments may be poor sources of bee‐associated bacteria. Decreased bee bread bacterial diversity may result in reduced function within hives. Although the dispersal of microbes is ubiquitous, this study has demonstrated landscape‐level effects on microbial community composition.

## 1 INTRODUCTION

Anthropogenic land use change is consistently threatening biodiversity, raising concerns about the consequences for ecosystem functioning (Ricketts et al., 2016). Considerable research has been undertaken to understand the linkages between ecosystem biodiversity, functioning, and services. Ecosystem function is not only determined by the phylogenetic diversity of its biota, but also by the functional traits of individuals, the distribution and abundance of these individuals, and their biological activity (Naeem & Wright, 2003). Geography and land use have further complex impacts on ecosystem function; for example, agricultural intensification in temperate habitats may lead to surface cooling from increased albedo, whereas in the tropics the opposite warming effect occurs due to reduced transpiration in crops and pastures compared with high‐biomass tropical forests (DeFries, Foley, & Asner, 2004).

Microbial impacts on plant–soil interactions are well documented (Macé, Steinauer, Jousset, Eisenhauer, & Scheu, 2016; Wardle et al., 1999). Microbial communities mediate key processes that control ecosystem nutrient cycling, and microbial function may therefore be key in determining plant ecosystem structure (Zak, Holmes, White, Peacock, & Tilman, 2003). Conversely, plant diversity also has complex effects on microbial communities and their ecosystem processes: Plant detritus biochemical composition limits the structure and function of microbial communities (Zak et al., 2003). Insects also maintain associations with diverse microbial communities for a range of functions, including resistance against colonization by pathogens (Lanan, Rodrigues, Agellon, Jansma, & Wheeler, 2016), degradation of toxins (Kikuchi et al., 2012), nutrient cycling (Dillon & Dillon, 2004; Engel & Moran, 2013), and tolerance of heat stress (Montllor, Maxmen, & Purcell, 2002).

The evolution of nest‐building and provisioning behavior in Hymenoptera has led to the storage of large amounts of food within the nest and facilitates increasing contact between nest‐mates and consequently within‐colony transfer of microbes (Kaltenpoth & Engl, 2014; Salem, Florez, Gerardo, & Kaltenpoth, 2015). Although this could result in a ubiquitous and homogeneous distribution of microbes throughout these nests, distinct microbiota clusters have in fact been observed in specific locations within Hymenopteran hives (Anderson et al., 2013). The microbial communities associated with food stores in these colonies are a combination of microbes that originate from forage and those derived from the host organism (Fewell & Bertram, 1999; McFrederick et al., 2012; Zasloff, 2017). For example, the gut (McFrederick et al., 2013), body surface (McFrederick et al., 2012), and hive infrastructure (Powell, Martinson, Urban‐Mead, & Moran, 2014) all contribute to this microbial community in honeybees.

Although microbial symbioses are typically thought of in terms of the singular benefits of specific members (Douglas, 1998; Montllor et al., 2002), it is likely that a broader community composition with multiple symbioses may be key to host fitness (Chandler, Morgan Lang, Bhatnagar, Eisen, & Kopp, 2011). For example, dysbiosis (the disruption of microbial community structure) can lead to increased disease susceptibility (Hamdi et al., 2011; Mattila, Rios, Walker‐Sperling, Roeselers, & Newton, 2012). Although triggers for dysbiosis in bees have not been identified, there may be a link between this and colony collapse disorder (CCD) in honeybees (Johnson, Evans, Robinson, & Berenbaum, 2009). CCD is likely related to a number of interacting factors including nutrition, pesticide exposure, land use change, and disruption of native microbial communities (Becher, Osborne, Thorbek, Kennedy, & Grimm, 2013).

Understanding dysbiosis in honeybees requires a prior knowledge of the identity of the native homeostatic microbial community and factors influencing its variability. Previous studies have used PCR‐based techniques to show that honeybees possess a core set of eight bacterial phylotypes that are observed in the guts of honeybees from the United States, Australia, South Africa, Germany, Sweden, and Switzerland (Jeyaprakash, Hoy, & Allsopp, 2003; Martinson et al., 2011; McFrederick et al., 2013; Mohr & Tebbe, 2006; Moran, Hansen, Powell, & Sabree, 2012; Olofsson & Vásquez, 2008). Members of the core gut community include *Snodgrassella alvi* (Betaproteobacteria: Neisseriales), *Gilliamella apicola, Frischella perrara* (Gammaproteobacteria: Orbales), Alphaproteobacteria, and Lactobacillae (Firmicutes: *Lactobacillaceae*). These eight phylotypes constitute 95% of bacterial 16S rRNA sequences cloned from within honeybee abdomens (Cox‐Foster et al., 2007; Moran et al., 2012; Olofsson & Vásquez, 2009).

Although studies specifically studying the gut microbiota suggest it may be highly conserved globally, other studies that have considered localized microbiomes within the hive (i.e., food stores, body surface, hive infrastructure) and different external environments bees are key in shaping the overall hive microbiome (Aizenberg et al. 2012). Honeybees transfer their microbiota horizontally within the hive and are exposed to nonhive microbes during foraging (McFrederick et al., 2012); both of these contribute to overall bacterial community composition within the hive.

The complex links between land use, floral diversity, and global hive microbial community lead us to hypothesize that the microbiota of bee bread may be linked to land use composition surrounding hives. Here, we explore this hypothesis with a 16S rRNA gene amplicon fingerprinting survey using both denaturing gradient gel electrophoresis (DGGE) and Illumina MiSeq next‐generation sequencing (NGS). NGS was used to gain a deeper understanding of species diversity across samples, while DGGE was used to provide a broader geographical context to community variation by analyzing a number of samples that would be prohibitively expensive for us to do with NGS. These latter data were then correlated against land use information from the UK Countryside Survey Land Cover Map (Morton et al., 2011). Finally, the use of DGGE and NGS methods within the same study allows for a useful comparison of the efficacy and comparability of these techniques.

## 2 MATERIALS AND METHODS

### 2.1 Study site and sample collection

Bee bread samples (*n* = 472) were collected from 29 honeybee (*A. mellifera*) hives within 23 apiary sites in North West England (Table S1) from 7th April to 2nd September 2012. Stratified sampling within hives (internal variation) and between hives (external variation) was used to partition variation in bee bread composition at different spatial scales. The hives in this study were structured in a nested fashion whereby honeycomb cells covered space on frames, and stored in (usually two) connected boxes which comprise a single hive (for further information see Donkersley, Rhodes, Pickup, Jones, and Wilson (2014) and Figure S1). The hives were owned by either hobbyist beekeepers or maintained as part of training suites for local beekeeping associations. Samples, consisting of whole intact individual cells of bee bread, were taken using a sterile sampling tool and placed into sterile 1.5‐ml microfuge tubes. Each hive visited three times over the program. Samples were immediately stored in Eppendorf tubes and frozen at −20°C within 2 hr of collection. A subset (*n* = 48) from 19 hives within 19 apiary sites were used in DNA sequencing with Illumina MiSeq due to constraints in the scope of this and a previous study (See Section 2.1, Table S1).

### 2.2 Sample processing

#### 2.2.1 PCR amplification

DNA was extracted from each of the 472 bee bread samples using the QIAamp DNeasy Plant Mini kit (Qiagen Ltd, Crawley, UK). DNA extractions were performed according to manufacturers’ specifications. Bacterial 16S rRNA genes were partially amplified by PCR using primer pair 515F (5′‐CCAGCAGCCGCGGTAA‐3′) and 806R (5′‐GGACTACCACGGTATCTAAT‐3′) (Relman, 1993), incorporating a 34‐bp GC clamp on the forward primer (Sheffield, Cox, Lerman, & Myers, 1989). PCR amplification was carried out using the Applied Biosystems Veriti thermal cycler (Thermo‐Fisher) in 20 μl volumes, such that each reaction contained the following: 2 μl (20 pmol) of each primer, 4 μl water (DNA‐free water; Sigma‐Aldrich Company Ltd, Poole, UK), 4 μl sample extracted DNA, and 10 μl Amplitaq Gold^®^ 360 Master Mix (Applied Biosystems, UK). Initial denaturation at 94°C for 3 min followed by 28 cycles of 94°C for 30 s, 53°C for 40 s, and 72°C for 60 s, with a final elongation step at 72°C for 5 min. PCR products were confirmed by agarose gel electrophoresis.

Assessment of bacterial diversity from these PCR products was incorporated from both denaturing gradient gel electrophoresis (DGGE) and Illumina MiSeq sequencing (NGS). The DGGE diversity index describes communities in terms of presence/absence of bands on a gel, which we delineate into operational taxonomic units (OTUs) based on unique positions (Figure S2), to provide broader data on bacterial richness. NGS was used to compliment these data by providing a higher resolution of community species diversity.

### 2.3 Denaturing gradient gel electrophoresis (DGGE)

Denaturing gradient gel electrophoresis was carried out using the Scie‐Plas TV400 vertical electrophoresis system (Scie‐Plas, Cambridge, UK). Electrophoresis was performed using 3 μl of each amplification product in polyacrylamide gels (6% polyacrylamide, 2% glycerol), with a denaturing gradient of 40%–65% (100% corresponding to 7 mol/L urea and 40% formamide). Gels ran at 60 °C, 20 V–30 mA for 10 min and then 100 V–30 mA for 1,250 V·Hrs (~16 hr) in 1× TAE buffer. Gels were subsequently stained with SYBR Gold (Invitrogen, Paisley, UK) for 30 min and visualized on a UV trans‐illuminator (320 nm). For DNA sequencing, each band of interest was excised from gels under UV light, using a sterile scalpel blade, and placed into individual sterile Costar Spin‐X centrifuge tube filters (Corning Inc., Tewksbury, USA) containing 40 μl 300 mmol/L sodium acetate. DNA was then extracted by centrifugation at 16,300 g for 10 min, and SYBR gold stain was removed by ethanol precipitation. The purified DNA (20 μl) was re‐amplified with primers 515F/806R as above.

The positions of bands on DGGE gels were normalized using a control sample and 10‐kb DNA ladder (Thermo‐Fischer Scientific, Paisley, UK) as an internal marker to permit comparisons between gels (Figure S2). Bacterial diversity in each sample was measured by counting the number of bands found in each lane, with the assumption that each band represents a different 16S rRNA gene sequence (and consequently a different bacterial genus) and therefore different operational taxonomic units (OTUs). A binary matrix was produced for each sample by noting the presence/absence of each OTU.

### 2.4 DNA sequencing and data processing

#### 2.4.1 Sanger sequencing of bands excised from DGGE gels

The DNA sequence of excised bands was determined (Beckman‐Coulter Sequencing; Essex, UK) by a single read on an ABI 3730XL Sanger sequencer, using the original primer 515F. Following elimination of chimeric or heteroduplex sequences using QIIME (http://qiime.org/index.html) via ChimeraSlayer (Caporaso et al., 2010), sequences were then aligned with those in the GenBank (ncbi.nlm.nih/genbank) database with the megaBLAST program (blast.ncbi.nlm.nih.gov; (Zhang, Schwartz, Wagner, & Miller, 2000).

#### 2.4.2 Illumina Sequencing

Illumina sequencing was carried out using a commercial facility at Molecular Research LP (http://www.mrdnalab.com, Shallowater, TX, USA) on the Illumina MiSeq platform using Illumina TruSeq DNA library preparation protocol for 2 × 250 bp paired‐end reads following the manufacturers’ guidelines, with 24 samples per lane.

Sequences obtained were first filtered by Phred quality scores using a standard Q25 20 bp window. Data processing were then performed in Mothur v. 1.36.1 (Schloss et al., 2009) based on the MiSeq SOP (Kozich, Westcott, Baxter, Highlander, & Schloss, 2013). Briefly, paired‐end sequences were merged using “*make.contigs*”; singleton and double sequences, those with ambiguous bases or shorter than 150 bp were removed with “*screen.seqs*” and chimeric sequences were removed using “*chimera.uchime*.” Sequences were clustered into OTUs using the “*dist.seqs*” and “*cluster*” functions.

Final OTUs were taxonomically classified using BLASTn (Altschul, Gish, Miller, Myers, & Lipman, 1990) against a curated international 16S rRNA database compiled from Ribosomal Database Project RDPII (https://rdp.cme.msu.edu), NCBI SRA (http://www.ncbi.nlm.nih.gov), and GreenGenes (http://greengenes.lbl.gov/) databases, maintained in propriety by MR‐DNA (Shallowater, TX, USA). Genus identifications were assigned to OTUs based on 97% similarity to reference sequences (DeSantis et al., 2006).

### 2.5 Sequence deposition

Sequences derived from DGGE‐PCR were deposited into GenBank and assigned Accession Numbers KF881801‐KF881848. Sequences derived from Illumina MiSeq sequencing were deposited on the NCBI Sequence Read Archive (http://trace.ncbi.nlm.nih.gov/Traces/sra) under accession numbers SRR1653719.

### 2.6 Statistical analyses

Analyses were performed using the *R* statistical software v3.1.1 (R Core Team, 2013). Spatiotemporal variation in the microbial community structure determined by DGGE was analyzed in a series of generalized linear mixed‐effects models (GLMMs) using the *lme4* package (Bates, Maechler, & Bolker, 2012). Significance values and approximate degrees of freedom were calculated using the “*lmerTest*” package (Kuznetsova, Brockhoff, & Christensen, 2013).

DGGE profiles for each sample were formatted as a binary matrix based on the presence/absence of each OTU. As such, to analyze these matrices, we used the number of OTUs in each sample as a response variable within a GLMM. The extent of internal variation at a nested hierarchy of spatial scales was analyzed according to methods described in Donkersley et al. (2014). These spatial scales (within‐frame, within‐hive box, within‐hive, and within‐apiary) were included as random effects in a GLMM. Significance of random effects was tested using stepwise deletion of random effects with chi‐squared tests on residual maximum likelihood estimates (following Zuur, Ieno, Walker, Saveliev, & Smith, 2009). These results were used to describe the degree of intrahive variance. The independent fixed effects tested included the geographical variables Eastings and Northings (*Eastings* & *Northings*), a temporal variable, Julian date (*Julian*: days since 1st January). The dependent variable in the model was total OTUs counts (Table 1).

**Table 1:** Variance components for DGGE OTU profiles in bee bread, for each of the hierarchical sampling levels (cells, frames, and boxes)

Between	*n*	Variance	*SD*	χ^2^	*p*
Cells	472	0.972	0.986	3.238	.072
Frames	83	0.847	0.92	0.405	.524
Boxes	43	8.896	2.983	8.829	.003
Residual	–	10.743	3.278	–	–

Variances and standard deviations (*SD*) indicate how variable OTU abundances are at different spatial scales. Random effects were tested using analysis of variance between models with sequential deletion of random variables using ML error structure as in Donkersley et al. (2014).

DGGE‐derived community data were analyzed for variation with environmental composition at three buffer zones around hives (500, 3,000, 10,000 m) using data from the UK Countryside Survey 2007 Land Cover Map (Morton et al., 2011). Briefly, 13 landscape cover types were analyzed, including improved grasslands, urban, broadleaf woodlands, and arable land covers (further details are described in Supplementary materials 1). Landscape cover composition was first analyzed by principal components analysis, producing six components for each of the buffer zones that explained 80%–90% of the variance (Table 2; Table S3). These components were then used as explanatory variables in a linear regression against DGGE‐derived bacterial community richness.

**Table 2:** Principal components analysis of landscape cover at 3,000 m surrounding the hives

Landscape type	Comp.1	Comp.2	Comp.3	Comp.4	Comp.5	Comp.6	Comp.7	Comp.8	Comp.9	Comp.10
Acid grassland	0.263	0.282	−0.294	−0.047	−0.197	−0.240	−**0.385**	**0.703**	0.108	0.001
Arable horticultural farmland	0.185	0.111	**0.531**	0.132	0.257	−0.249	−0.038	0.078	−0.063	−0.065
Broadleaf woodland	**0.336**	−0.035	0.192	−**0.594**	−0.183	−0.180	**0.319**	0.015	**0.313**	0.036
Urban	0.290	**0.365**	−0.172	0.114	**0.316**	0.297	0.186	−0.032	0.102	0.030
Coniferous woodland	**0.374**	0.014	−0.085	−**0.517**	0.087	0.117	−**0.379**	−**0.302**	−**0.536**	−0.113
Dry scrub heath	0.290	**0.365**	−0.172	0.114	**0.316**	0.297	0.186	−0.032	0.102	0.030
Freshwater	0.185	0.111	**0.531**	0.132	0.257	−0.249	−0.038	0.078	−0.063	−0.065
Improved grassland	0.123	−**0.487**	0.015	0.157	**0.342**	0.187	−**0.403**	0.191	−0.038	0.293
Littoral rock	−**0.341**	0.253	0.065	−0.276	0.267	−0.025	−**0.504**	−0.272	**0.575**	0.072
Littoral sand	−**0.344**	0.251	0.201	−**0.312**	0.061	0.191	0.163	0.298	−**0.310**	**0.651**
Neutral grassland	−0.068	−**0.435**	−0.138	−**0.324**	**0.502**	0.049	0.267	**0.339**	0.165	−0.233
Rough grassland	0.181	−0.122	**0.410**	0.008	−**0.374**	**0.688**	−0.132	0.133	0.256	−0.083
Semi‐littoral sands	−**0.392**	0.244	0.119	−0.118	0.066	0.220	−0.047	0.260	−0.229	−0.633
Variance	2.10	1.74	1.58	0.92	0.89	0.73	0.59	0.49	0.40	0.28
% Explained	21.56	17.85	16.24	9.49	9.16	7.54	6.07	5.04	4.13	2.92
Cumulative % explained	21.56	39.41	55.65	65.14	74.30	81.84	87.91	92.95	97.08	100.00

The factor loadings for each landscape type to each principal component are given, factors >0.3 are bold, for full factor loadings of the other buffer zone sizes see Table S3.

Data derived of Illumina MiSeq sequencing including OTU counts, Shannon and Simpsons diversity were calculated using *diversity* function in the “vegan package” (Oksanen et al., 2013). To determine similarities between data total OTU abundance generated using DGGE and NGS, matched samples where both data exist were compared using a Pearson's correlation test. Next, we examined the genera found in both DGGE and NGS across the whole study and finally calculated the “diversity differential” within each sample; for example, the number of extra genera NGS was capable of detecting (relative to the total diversity detected).

## 3 RESULTS

### 3.1 Overall assessment of bacterial diversity

DGGE was used for all samples only to provide a broad comparison of bacterial community richness. PCR amplification of the V4 region of the 16S rRNA gene from 472 bee bread samples and separation by DGGE resulted in a total of 73 different bands (OTUs). Each bee bread sample comprised on average of 6.16 ± 4.14 OTUs (mean ± *SD*). All 73 bands were excised from DGGE gels and successfully re‐amplified and sequenced, resulting in partial 16S rRNA gene sequences ranging between 241 and 286 bp in length (Table S2). Sequence alignments correctly identified a total of 10 distinct bacterial genera, although some of the identity scores were <95%, which limited the resolution of some OTUs. These genera were as follows: *Acinetobacter*,* Arsenophonus*,* Bacillus*,* Clostridium*,* Enterobacter*,* Erwinia*,* Frischella*,* Lactobacillus*,* Massilia*,* Phyllobacterium*,* Pseudomonas*,* Raoultella*,* Rosenbergiella*. Two OTUs (BB20 and BB21) shared closest homology with the genus *Acinetobacter* and three with *Lactobacilli* (BB7, BB12, and BB16). Four OTUs were *Enterobacter* (BB5, BB38, BB49, and BB51), and four were *Pseudomonas* (BB19, BB22, BB28, and BB30). These four bacterial genera occurred in up to 18.9% (*Lactobacillus*), 12.7% (*Acinetobacter*), 12.6% (*Pseudomonas*), and 12.5% (*Enterobacter*) of samples.

Based on the degree of intersample variation within DGGE results, we selected a subset of 48 bee bread samples that were most distinct (i.e., outside of the interquartile range of DGGE‐derived diversity data) for further analysis in Illumina MiSeq sequencing. This technique was used to gainer a deeper understanding of species diversity across samples.

#### 3.1.1 Sequencing quality control

Sequencing of amplified DNA from bee bread generated 7,103,296 raw reads with an average read length of 272 bp (271–273 bp). Post filtering, 2,470,158 reads were clustered to 3729 distinct OTUs.

#### 3.1.2 Bacterial Phyla

**Figure 1:**
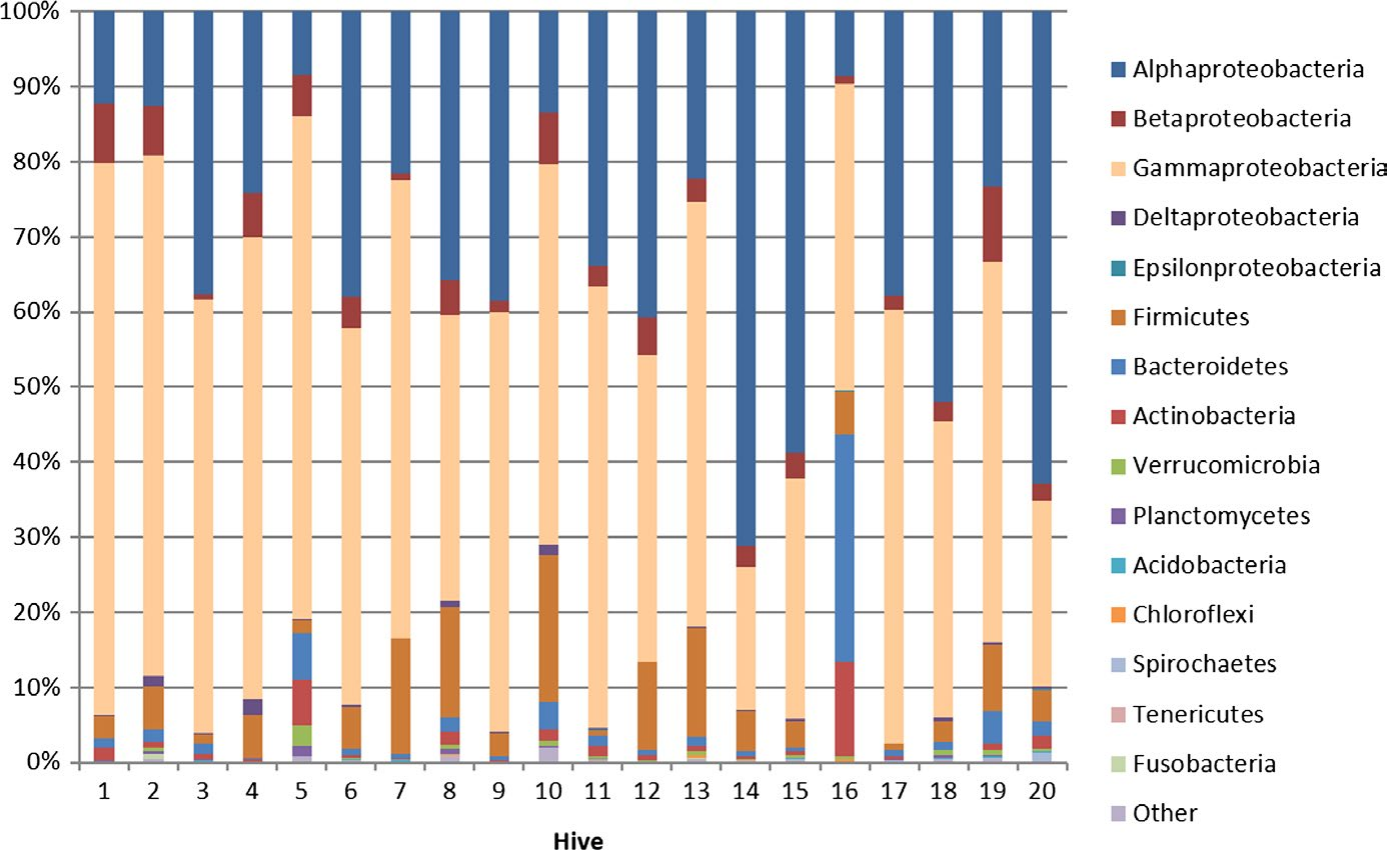
Phylum‐level distributions of bacterial community, determined by Illumina MiSeq sequencing of bee bread from 20 hives, organized by location (on an east–west axis left to right)

**Figure 2:**
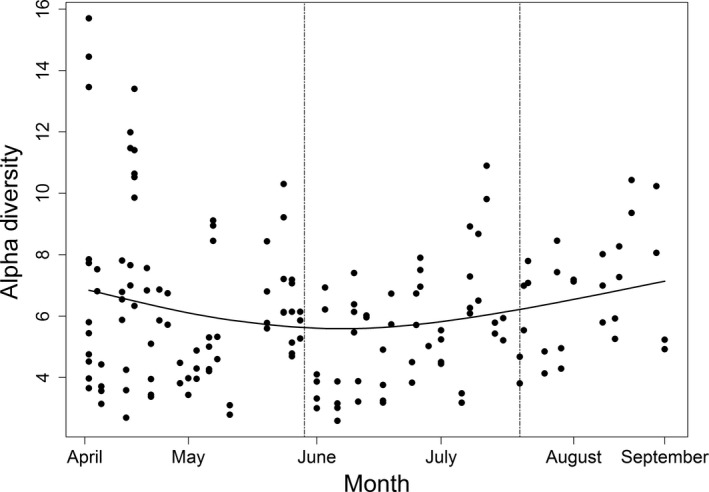
Temporal variation in bacterial community composition of bee bread determined by 16S rRNA gene PCR‐DGGE. Fitted data from minimal models showing temporal variation in alpha diversity

OTUs were clustered into 24 bacterial phyla with Proteobacteria representing more than 80% of all the phyla in bee bread samples (Figure 2). All bee bread samples harbored diverse lineages of bacterial phyla, comprising on average 13 phyla (mean ± *SD*: 12.8 ± 3.2, range: 6–20) with the top 5 most relatively abundant being *Bacteroidetes*,* Firmicutes*,* Alpha‐proteobacteria*,* Beta‐proteobacteria,* and *Gamma‐proteobacteria* (Figure 1).

#### 3.1.3 Bacterial genera

Across the 24 phyla, 581 distinct bacterial genus identities were assigned to the sequence OTUs. Each sample of bee bread comprised on average 96 bacterial genera (mean ± *SD*: 96.7 ± 40.2, range: 43–215). The five most common genera in terms of sequence reads were as follows: *Pseudomonas* (32.4%), *Arsenophonus* (13.0%), *Lactobacillus* (8.2%), *Erwinia* (7.7%), and *Acinetobacter* (5.2%), which in total accounted for 66.48% of the sequences generated within this study. Eleven genera were present in all 48 samples, including *Pseudomonas*,* Arsenophonus*,* Orbus*,* Lactobacillus*,* Erwinia,* and *Acinetobacter*, although the *Saccharibacter*,* Raoultella*,* Tatumella*,* Massilia,* and *Sphingomonas* accounted for less than 2% of sequences in total.

### 3.2 Comparison of bacterial community composition using DGGE and NGS

Across the 48 samples that we applied both DGGE and NGS, we observed clearly different estimates of bacterial community composition. NGS reported approximately 16 times more OTUs compared with DGGE (Figure S4) and therefore provides a deeper understanding of species diversity. Paired analysis of total bacterial diversity showed no significant correlation between the DGGE and NGS (*r* = .067, *t* = 0.458, *df* = 46, *p* = .649). Importantly, each of the 10 genera detected by DGGE was all found within the NGS data, although they were detected in different proportions (Table S4). The diversity of genera found by NGS but not by DGGE in each sample (relative to the number of genera found by NGS) was examined and was highly conserved across all samples. The mean “diversity differential” between NGS and DGGE was 0.899 ± 0.040 (range: 0.771–0.959). The low standard deviation of the diversity differential indicates that the greater power of NGS is not greatly different between samples.

### 3.3 Intrahive variation of bacterial communities (DGGE)

The hives in this study were structured in a nested fashion, whereby each hive contains one or two boxes, each containing eight‐twelve frames. These frames are covered in >300 cells, subsequently allowing for partitioning of variance components. In terms of OTU abundance, significant variation between boxes was observed within the same hive by DGGE. More specifically, cells of bee bread located within different boxes had significantly different OTU abundances, not matched by inter‐ and intraframe variation (Table 1). This shows when comparing cells from the same frames, each individual cell is unique, but this averages away when the whole frame is considered.

### 3.4 Spatiotemporal variation of bacterial communities (DGGE)

The broader scope of DGGE data allows us to provide a broader geographical analysis of bacterial community variation. There was a significant quadratic seasonal relationship in total OTU abundance (GLMM: *Day + Day* ^2^: *b1 *±* SE* = −27.749 ± 15.211, *F*_1, 464_ = 4.262, *p* = .042; *b2 *±* SE* = 0.300 ± 0.100, *F*_1, 464_ = 7.750, *p* = .011, Figure 2). OTU abundance varied with hive location, with significant diversity increases in Eastern hives (*Eastings*:* b *± *SE* = 0.619 ± 0.119, *F*_1, 464_ = 8.227, *p* = .004), but not across the Northings axis (*F*_1, 464_ = 1.309, *p* = .524).

### 3.5 Landscape composition and bacterial community (DGGE)

**Figure 3:**
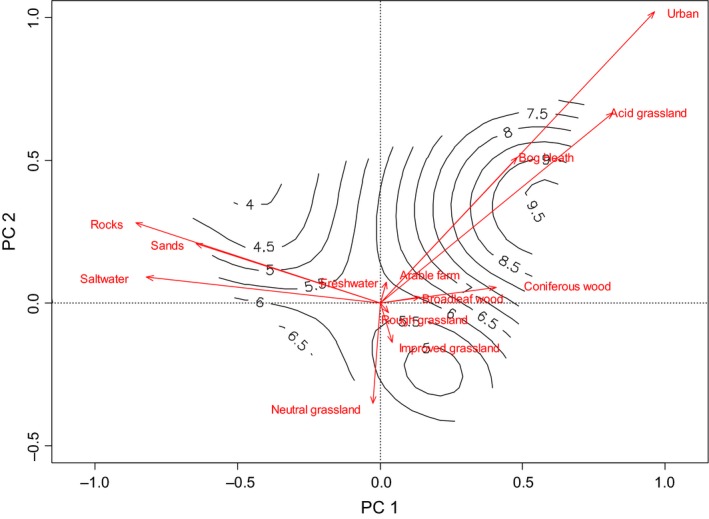
Principal components biplot of landscape cover composition at 3000 m (using axes 1 and 2 from the PCA); arrows indicate the loading of each landscape type. Surface plot indicates the number of OTUs (i.e., the diversity) detected by DGGE within each sample

Estimates of bacterial community richness were correlated with local land use composition determined by PCA at all three buffer zone sizes (Figure 3, Table 3). At the 500 m buffer zone, community richness was negatively correlated with PC1 (improved grassland and urban) and PC2 (freshwater and broadleaf woodland). Richness was also positively correlated with PC3 (acid grassland and rough grassland). At the 3,000 m buffer zone, we found positive correlations between community richness and PC1 (semilitoral sands and broadleaf woodland) and PC4 (broadleaf woodland and coniferous woodland). Here, richness was also negatively correlated with PC3 (arable horticultural farmland and freshwater) and PC5 (freshwater and rough grassland). Finally, at the 10,000 m buffer zone, bacterial richness was negatively correlated with PC2 (improved grassland and urban) and positively correlated with PC3 (litoral sand and semilitoral sands).

**Table 3:** Bacterial community richness (DGGE) modeling results with landscape composition estimated by principal components analysis (PCA)

Buffer zone size		Factor 1	Factor 2	Factor 3	Estimate	SE	t	*p*
500 m	(Intercept)	–	–	–	5.774	0.180	32.099	<.001
	PC1	Improved grassland	Urban	Littoral sand	−0.143	0.086	−1.665	.097
	**PC2**	**Freshwater**	**Broadleaf woodland**	**Rough grassland**	−**0.333**	**0.103**	−**3.220**	**.001**
	**PC3**	**Acid grassland**	**Rough grassland**	**Broadleaf woodland**	**0.327**	**0.116**	**2.812**	**.005**
	PC4	Littoral sand	Fresh water	Urban	0.048	0.160	0.301	.764
	PC5	Neutral grassland	Littoral sand	Neutral grassland	0.012	0.179	0.067	.947
	PC6	Broadleaf woodland	Acid grassland	Fresh water	−0.194	0.256	−0.758	.449
3,000 m	(Intercept)	–	–	–	5.774	0.178	32.392	<.001
	**PC1**	**Semilitoral sands**	**Broadleaf woodland**	**Coniferous woodland**	**0.167**	**0.085**	**1.971**	**.049**
	PC2	Improved grassland	Neutral grassland	Urban	0.150	0.103	1.462	.145
	**PC3**	**Arable horticultural farmland**	**Fresh water**	**Rough grassland**	−**0.322**	**0.113**	−**2.858**	**.004**
	**PC4**	**Broadleaf woodland**	**Coniferous woodland**	**Neutral grassland**	**0.386**	**0.193**	**2.000**	**.046**
	**PC5**	**Neutral grassland**	**Rough grassland**	**Improved grassland**	−**0.669**	**0.200**	−**3.344**	**.001**
	PC6	Rough grassland	Urban	Dry scrub heath	−0.183	0.243	−0.754	.451
10,000 m	(Intercept)	–	–	–	5.774	0.178	32.372	<.001
	PC1	Rough grassland	Broadleaf woodland	Acid grassland	−0.143	0.085	−1.679	.094
	**PC2**	**Improved grassland**	**Urban**	**Coniferous woodland**	−**0.333**	**0.102**	−**3.247**	**.001**
	**PC3**	**Littoral sand**	**Semilittoral sands**	**‐**	**0.327**	**0.115**	**2.836**	**.005**
	PC4	Littoral rock	Fresh water	Improved grassland	0.048	0.158	0.304	.762
	PC5	Coniferous woodland	Dry scrub heath	Acid grassland	0.012	0.178	0.067	.946
	PC6	Arable horticultural farmland	Improved grassland	Urban	−0.194	0.254	−0.764	.445

The three greatest factors (>0.3 factor loading, see Table 2) for each component in the PCA are indicated, with further details on each component available in Table S3. Bold values indicate statistically significant (*P* < 0.05) correlation with bacterial richness.

## 4 DISCUSSION

Microbial communities are key to ecosystem processes that control nutrient cycling, both on a broad environmental scale and on a narrow host‐organism scale. We studied the bacterial community using a combination of DGGE and Illumina MiSeq. Illumina was used to gain a deeper understanding of precise community composition of a subset of samples. DGGE was applied to a larger number of samples where the costs of MiSeq had become prohibitive and therefore allowed us to study a greater number of bee breads across broader geographical axes. This is the first study to link estimates of land use (grassland, woodland, urban, etc.) with honeybee bacterial community diversity.

### 4.1 Bacterial community composition

Illumina MiSeq DNA sequencing was used to give a deeper estimate the species diversity of the bee bread bacterial microbiome and demonstrated that bee bread food stores from honeybee hives comprise a diverse array of bacteria, primarily from the genera *Pseudomonas*,* Acinetobacter,* and *Lactobacillus*. Through examination of these data, a core bacterial microbiome (a set of taxa present in all samples) emerges, represented by members of the Enterobacteriaceae, Lactobacillaceae, Pseudomonadaceae, Comamonadaceae, Oxalobacteraceae, and Sphingomonadaceae. This community is similar to previous research into the gut microbiome of honeybees (Anderson et al., 2013).

Based on Illumina MiSeq data, *Pseudomonas*,* Arsenophonus*,* Lactobacillus*,* Erwinia,* and *Acinetobacter* were the most common genera detected. Of these, *Acinetobacter* and *Lactobacillus* were also the most common genera that could also be confidently identified from sequences found by DGGE. *Acinetobacter, Lactobacillus,* and *Enterobacter* have previously been detected in the digestive systems of honeybees by both molecular and culture‐based methods (Kaznowski et al., 2005; Vásquez & Olofsson, 2009; Vasquez et al., 2012).

Members of the Firmicutes (*Lactobacillus*), Enterobacteriales (*Enterobacter*), and Bifidobacteriales (*Actinobacteria*) have also been found within the floral nectaries and surfaces of pollen grains of insect‐pollinated plants (Ambika Manirajan et al., 2016; Lenaerts et al., 2016). Curiously, within the insect‐pollinated plants, different species may have “signature” microbial communities (Ambika Manirajan et al., 2016), which are combined with the gut and hive microbiomes to form the community found associated with bee bread here.

An understanding of the distinctions between symbiosis, commensalism, and parasitism is limited by both the nature of data derived from sequencing studies and our knowledge of the functional roles of these microorganisms. Many potential roles of the *Enterobacter, Lactobacilli,* and *Acetobacter* within the bee gut microbiome have been suggested. Our study instead focused on the bacterial microbiome of bee bread, which is comparatively poorly understood. The most abundant family found in bee bread in the present study, the Enterobacteriaceae, is large and includes many animal‐ and plant‐associated bacteria, found as free‐living associates of many insects (Chandler et al., 2011). Enterobacteriaceae are commonly found on the surface of pollen grains (Ambika Manirajan et al., 2016), and some lineages within the Enterobacteriaceae have demonstrated antibiotic activity specifically against honeybee pathogens (Kaltenpoth & Engl, 2014).

*Orbus* (family Pasteurellaceae) have also been previously detected using molecular methods in the gut microbiome of honeybees (Ahn et al., 2012). *Orbus* species are most abundant in the guts of many species of in fruit‐ and flower‐feeding insects (Chandler et al., 2011). We found them in all samples of bee bread; significant increases in the abundance of *Orbus* species are associated with high protein, low carbohydrate diets (Chandler et al., 2011). These organisms may have an important role in the high protein environment of bee bread (Donkersley et al., 2014).

*Gilliamella*,* Erwinia,* and *Frischella* (Order: Enterobacteriales) were abundant in bee bread and have been found associated with honeybee guts (Alexandrova et al., 2002; Engel, Kwong, & Moran, 2013). *Snodgrassella alvi* and *Gilliamella apicola* are key members of the core gut microbiome of honeybees (Powell et al., 2014) and demonstrate complimentary metabolic pathways for the metabolism of carbohydrates (Lee, Rusch, Stewart, Mattila, & Newton, 2015). The role of bacteria in altering the nutritional content of bee bread has recently been questioned, and these organisms may have alternative functions (Anderson et al., 2014). *S. alvi* and *G. apicola* may protect bees from opportunistic infections, but this effect depends on the age of bee bread, its bacterial community composition and host fitness (Maes, Rodrigues, Oliver, Mott, & Anderson, 2016).

*Frischella perrera* is an opportunistic pathogen that (under high abundances) causes symptoms parallel to emerging models of dysbiosis, such as *Clostridium difficile* in humans (Buffie et al., 2012). *S. alvi* and *G. apicola* from bee bread may protect from *F. perrera* dysbiosis by early establishment of a stable gut microflora (Maes et al., 2016). Likewise, bumble bee workers that have reduced abundances of the typical *S. alvi* and *G. apicola* have a greater chance of hosting enteric pathogens (Cariveau, Powell, Koch, Winfree, & Moran, 2014).

Illumina MiSeq sequencing also detected other major bacterial genera commonly found in the gut microbiome of bees: *Saccharibacter*,* Raoultella*,* Tatumella*,* Massilia,* and *Sphingomonas*. These bacteria may be commensal within the honeybee gut, as no specific function has been identified yet (Babendreier, Joller, Romeis, Bigler, & Widmer, 2007; Jeyaprakash et al., 2003).

### 4.2 Variation in community composition

DGGE was used to analyze spatiotemporal variation in bee bread bacterial community composition and correlate this with land use composition. Our results broaden the scope of previous studies by examining the bacterial community associated with bee bread, which may be a key source for many gut symbionts (Anderson et al., 2014; Maes et al., 2016). We demonstrate that this bacterial community varies significantly in its composition spatially at different scales, both internal (within the hive) and external (between hives).

#### 4.2.1 Internal variation

The nests of social insects are structured based on the rearing of young and long‐term storage of nutrient‐rich material. In recent studies of ants and honeybees, the microbial communities associated with different castes and nest components were shown to vary more by component than by species (Grubbs et al., 2015; Ishak et al., 2011; Scott et al., 2010). Our results here suggest an even finer level of internal microbiome variation than these studies. Here, we found statistically significant variation in the bacterial community composition of bee bread between the same hive components (in this case bee bread cells) located in different boxes within a hive.

Internal spatial variation in bacterial community composition has been previously observed between larval cells on the same frame in honeybee hives (Powell et al., 2014). The extent of cup‐to‐cup variation is consistent with social transmission of many of these bacteria, where cell‐to‐cell differences in bee breads and larval cups are most likely caused by exchange of bacteria from adults to newly emerged workers within the hive (Grubbs et al., 2015; Powell et al., 2014). Our results suggest that bee bread communities may have distinct origins in different boxes, but similar origins within them, due to spatial compartmentalization of tasks by specific groups of bees. Different insect pollinator species have distinct “signature” microbial communities, although individual honeybees are too small to impact the overall community (Ushio et al., 2015); here, we reveal the effect of groups on detectable bacterial community composition.

Conversely, our findings of box‐to‐box differences suggest that honeybees may be dependent on their environment rather than their hive mates for microbial communities. Production of bee bread is dependent on multiple plant species pollens and preparation by nurse bees (Camazine et al., 1998; Di Pasquale et al., 2013). We could not determine whether bee gut microbiota or floral nectaries are more influential on microbial community composition (Anderson et al., 2013). Our results do allow us to hypothesize that the bacterial community composition of bee bread is derived from the environment. To confirm this hypothesis however, future studies must determine the origin of the bacteria found in bee bread by surveying their potential environmental sources.

#### 4.2.2 External variation

The bacterial community of bee bread varied nonlinearly through the year and spatially across the study site. Previous research indicates that microbial species richness may vary through the year (Mattila et al., 2012). Temporal changes to microbial communities can lead to increased disease susceptibility in honeybees (Hamdi et al., 2011; Maes et al., 2016; Mattila et al., 2012). The microbial community species richness in bee breads from our study exhibited a minimum in mid‐June. Peaks in pathogen abundance (i.e., Nosema, Crithidia, or bee viruses) have been demonstrated in other studies during mid‐summer (Runckel et al., 2011). However, we could not directly attribute this to reduced bacterial diversity as our study did not specifically test for pathogens observed previously.

Bee bread bacterial community composition varied significantly with hive location. Hives in the east of the study area hosted demonstrably greater bacterial diversities measured by DGGE. Environmental factors have previously been shown to play a key role in determining the nutritional composition of the diet bees is producing (Donkersley et al., 2014). The spatial variation observed here suggests these effects may extend to the bacterial community within bee bread as well. Although floral nectaries and pollen stamens are typically dominated by Proteobacteria, Firmicutes, and Actinobacteria, insect‐pollinated plant species possess a less diverse pool of microbes in comparison with the wind‐pollinated ones, suggesting a levelling effect by insect vectors (Alvarez‐Perez, Herrera, & de Vega, 2012; Ambika Manirajan et al., 2016; Lenaerts et al., 2016). Microbial communities vary significantly between plant species, and these bacterial communities could vary with land use composition, which could consequently influence the diversity in bee bread.

#### 4.2.3 Land use

By studying data from the Countryside Survey Land Cover map (Carey et al., 2008), we found significant correlations between bacterial composition and landscape community. Specifically, positive correlations were found with coastal landscape types (littoral rock and sand) and negative correlations with improved grasslands and coniferous woodland. Agriculturally improved grasslands are associated with reduced floral diversity (Tallowin, Smith, Goodyear, & Vickery, 2005), yet have been suggested to be of high nutrient value to pollinators nationally due to the relative high abundance of white clover (Baude et al., 2016). We suggest that the reduced floral diversity in improved grasslands may be driving a reduction in bacterial community diversity; a similar pattern has been observed in soil microbial communities (Macé et al., 2016). Conversely, natural grasslands such as acidic and rough grassland types typically host a high diversity of rare plant species (Pykälä, Luoto, Heikkinen, & Kontula, 2005), which in turn provide better support for pollinator communities (Orford, Murray, Vaughan, & Memmott, 2016; Ward & Wilby, 2015). This superior pollinator recruitment was correlated here with an increasing diversity of bacterial community (Table 3), potentially linking forage diversity with microbial recruitment.

The high diversity of exotic introduced garden species associated with urban environments represents a diverse source of pollen for bees, which may result in increased nutrition (Donkersley et al., 2014). Bee diversity is strongly affected by plant diversity in urban environments (Bates et al., 2011). Decreased bacterial diversity in bee breads associated with urban environments (Table 3) suggests that the increased diversity of non‐native plants could provide be impacting bees’ ability to recruit diverse microbiota. Insect pollinators are constrained by inhabiting environments populated by a microbiome with which they have coevolved (Zasloff, 2017). As urban environments produce less diverse bee breads, this may be evidence that bees suffer foraging on a community of non‐native plants that they have not coevolved with. Hence, this may have negative consequences for bee fitness (Zasloff, 2017). Further study of the relationship between plants in non‐native environments and microbial diversity of their nectaries could usefully explore this interaction.

Bacterial community diversity was also found to be positively correlated with increasing broadleaf woodland cover. This is also linked to both increased protein content and floral diversity in bee bread (Donkersley et al., 2014, 2017). Although it is commonly believed that bacterial diversity is a benefit to bees (Ambika Manirajan et al., 2016; Lenaerts et al., 2016; Tian, Fadhil, Powell, Kwong, & Moran, 2012), direct measures of functional community contributions to bee survival are limited. Within the honeybee gut, studies indicate that species diversity is low, while strain diversity is high (Moran, 2015). Strain diversity potentially provides more metabolic functions that benefit hosts than species diversity; for example, *G. apicola* strains vary in ability to process carbohydrates (Lee et al., 2015).

Given the evidence for a beneficial role of the gut microbiota and the role bee bread plays in influencing this community, effects that interfere with normal microbiota are likely to be detrimental (Maes et al., 2016). We know microbial communities associated with bee bread are at least partially determined by floral nectaries (Alvarez‐Perez et al., 2012), and bee bread itself is a major contributor to the hive microbiome (Fewell & Bertram, 1999; McFrederick et al., 2012). We therefore theorize that loss of diversity could lead to effects similar to microbial dysbiosis (Hamdi et al., 2011; Maes et al., 2016; Mattila et al., 2012). Rather than the direct effects on microbial communities caused by widespread application of antibiotics in the United States (Tian et al., 2012), our study suggests land use change may also be having an indirect detrimental effect on the microbiota of bee bread. Since bee bread effects the ability of bee gut microbiome to resist infection by opportunistic pathogens (Tian et al., 2012), we therefore believe could be an indirect link between landscape composition and bee fitness.

### 4.3 Sequencing technology

The technologies used in this study gave different estimates of bacterial community composition, with NGS reporting approximately 16 times more OTUs as DGGE. DGGE did not detect genera that the NGS also could not detect, which is explained by the deeper resolution of sequencing that NGS sequencing returns compared with traditional PCR‐based methods of DGGE (Schwartz, Oren, & Ast, 2011). These two techniques also produce different estimates for community composition (DGGE and NGS producing OTU counts) due to the ability of the latter to detect low abundance DNAs and the somewhat random nature of DNA amplification under DGGE conditions. We also found that the deeper sequencing offered by NGS was finding a greater number of bacterial genera that was consistent across all samples. Consequently, this enables us to suggest that although DGGE can only detect a small subset of the bacterial community. This consistency allows us to draw meaningful comparisons between the two techniques and across our sampling program. Furthermore, we also examined the “diversity differential” between the two techniques, which we defined here as the number of genera detected by NGS, but not DGGE. The fact that NGS detects more genera than DGGE, but this is evenly distributed across our samples means this power is evenly applied across all samples. These findings combined justify our inclusion of both DGGE and NGS within this study and allow us to suggest that DGGE usefully analyses a consistent subset of the microbial community.

NGS also offers estimates of relative abundances of these OTUs and allows for more complex assessments through ecological diversity indices (Mattila et al., 2012). Clearly, the depth of analysis possible from next‐generation sequencing technologies allows for a more complete analysis of bacterial community composition. However, despite the lack of complex comparable indices derivable from DGGE, the relative low costs of this technology allowed us to implement an analysis of bacterial communities across a broader spatiotemporal scale where the costs of next‐generation sequencing approaches would have to be prohibitive (Joossens et al., 2011; Machtelinckx et al., 2012; Shimano, Sambe, & Kasahara, 2012). Thus, both of these techniques maintain merit for discussing microbial ecology.

Issues with DNA sequencing must be acknowledged for both classical PCR and Illumina MiSeq sequencing (NGS). For DGGE‐analysis, these include nonequitable amplification, sequencing error and insufficient sequence length for accurate species identification. For NGS, errors of most concern occur in the bioinformatics pipeline (Lee et al., 2012; Zhou et al., 2011). Biases such as mutation and chimeras may cause overestimation of richness, while primer mismatching and others lead to underestimation (Wang et al., 2012). We are confident that our data processing methods (see materials and methods) allow us to objectively examine microbial community composition. The read length used in this study (<300 bp) was used to assign OTUs to genus level and may underestimate species richness.

This work contributes to a broader body of research on biodiversity effects on microbial ecosystem function. Future research could usefully explore how the bacterial communities derived from forage plants and transmitted horizontally in the hive interact and combine within the hive to determine a stable community capable of providing bees with support in digesting pollen and protecting against pathogens. Perhaps a combined survey of the microbial communities of locally available floral nectaries, the gut microbiota of bees and microbiome of bee bread could partition these sources.

## CONFLICT OF INTEREST

None declared.

## AUTHOR CONTRIBUTIONS

PD, GR, RWP, and KW conceived and designed the experiments. PD performed the experiments. PD analyzed the data. All other authors contributed to writing the manuscript.
