# Supplementary material for: Bacterial communities associated with honeybee food stores are correlated with land use
Source: Ecol Evol. 2018 Apr 16;8(10):4743–56. doi: 10.1002/ece3.3999 (PMC5980251; doi:10.1002/ece3.3999)
Supplement: Supplementary file 1 [file ECE3-8-4743-s001.docx]

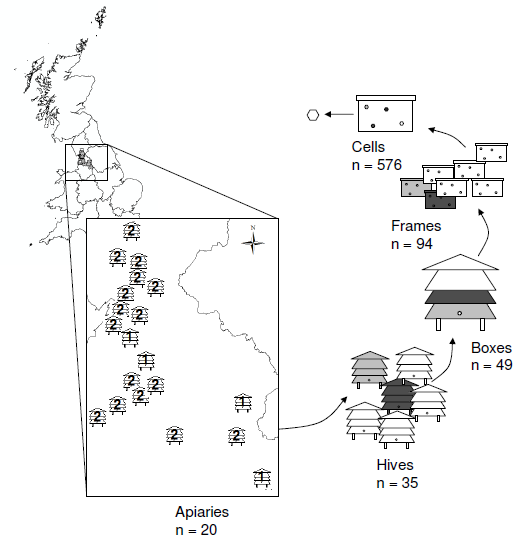


**43**

**83**

**4722**

**23**

**29**

Figure S1. Schematic diagram of the stratified sampling technique used to sample apiaries in
the northwest of England. The location of apiaries (n = 23) is highlighted by the hive drawings. In total, 472 cells were sampled for beebread, which were obtained from 83 frames, held in 43 boxes from 29 hives across the 23 apiaries.
