# Supplementary material for: Bacterial communities associated with honeybee food stores are correlated with land use
Source: Ecol Evol. 2018 Apr 16;8(10):4743–56. doi: 10.1002/ece3.3999 (PMC5980251; doi:10.1002/ece3.3999)
Supplement: Supplementary file 2 [file ECE3-8-4743-s002.doc]

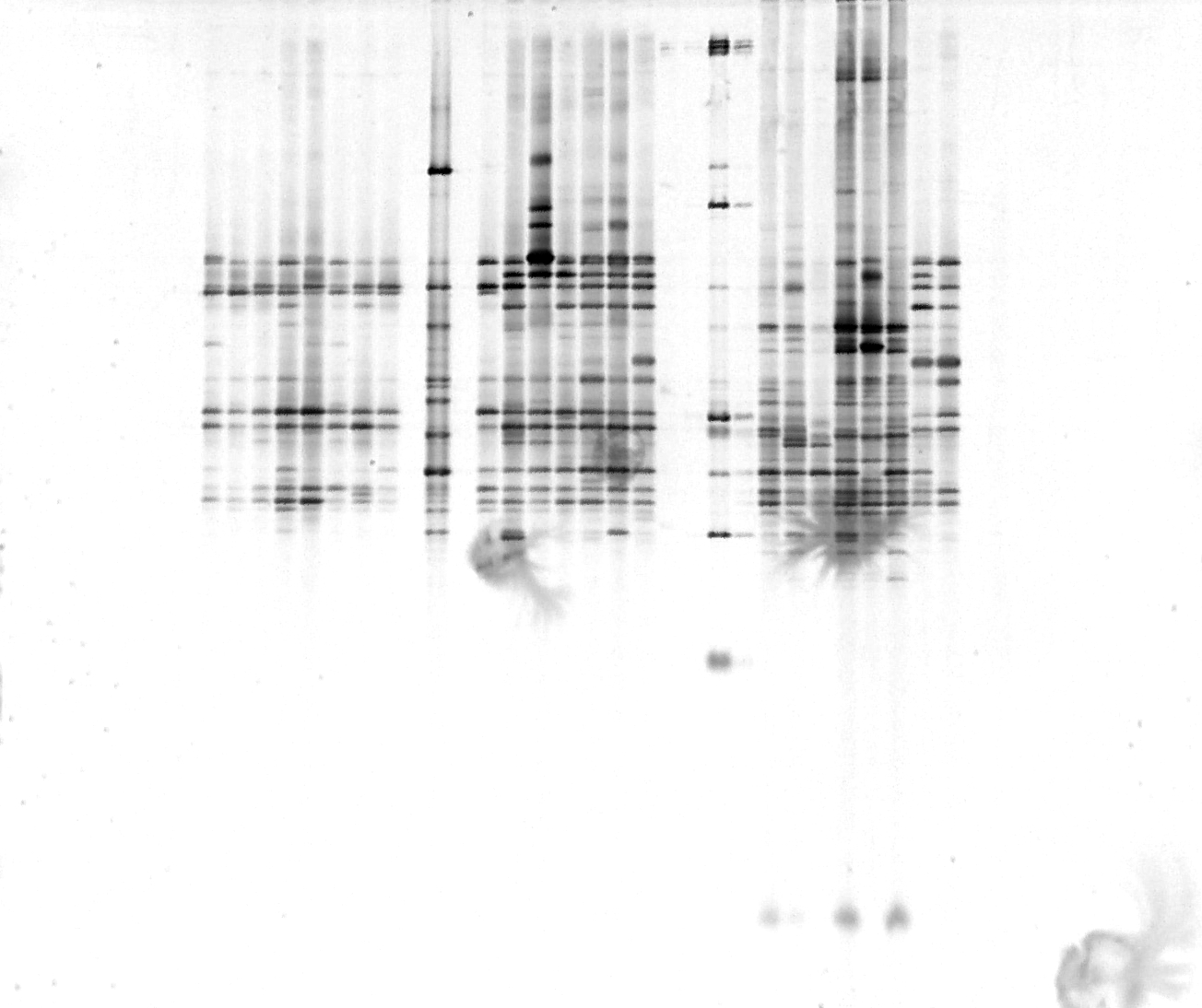


M

L

1

2

3

4

5

6

7

8

9

10

11

12

13

14

15

L

15

M

1

2

3

4

5

6

7

8

9

10

11

12

13

14

L

15

M

1

2

3

4

5

6

7

8

9

10

11

12

13

14

L

15

M

L

1

2

3

4

5

6

7

8

9

10

11

12

13

14

15

L

15

**Figure S2. Section of a DGGE profile of PCR-amplified partial 16S rRNA bacterial genes.** Corresponding marks indicate how bands were identified and aligned. Numbers 1-15 indicate samples of bee bread; M: internal DGGE marker, L: 10kb DNA marker.
