# Supplementary material for: Bacterial communities associated with honeybee food stores are correlated with land use
Source: Ecol Evol. 2018 Apr 16;8(10):4743–56. doi: 10.1002/ece3.3999 (PMC5980251; doi:10.1002/ece3.3999)
Supplement: Supplementary file 3 [file ECE3-8-4743-s003.doc]

Figure S3. Spatial plots of bacteria diversity derived from next generation sequencing data. Spatial patterns of diversity indices are calculated and plotted using “thin plate splines” function in the *fields* package.
