# Supplementary material for: Bacterial communities associated with honeybee food stores are correlated with land use
Source: Ecol Evol. 2018 Apr 16;8(10):4743–56. doi: 10.1002/ece3.3999 (PMC5980251; doi:10.1002/ece3.3999)
Supplement: Supplementary file 4 [file ECE3-8-4743-s004.doc]

**Figure S4.** The diversity of bacterial genera found in bee bread; (a) OTU abundance from Illumina MiSeq (x̅ ± SD [n] = 96.71 ± 40.15 [48]) and from DGGE (6.16 ± 4.14 [48]). (b) Species accumulation curves of Illumina MiSeq (red) and DGGE (blue) data.
