# Supplementary material for: Bacterial communities associated with honeybee food stores are correlated with land use
Source: Ecol Evol. 2018 Apr 16;8(10):4743–56. doi: 10.1002/ece3.3999 (PMC5980251; doi:10.1002/ece3.3999)
Supplement: Supplementary file 5 [file ECE3-8-4743-s005.doc]

Table S1. Locations of hives sampled during the study for denaturing gradient gel electrophoresis (n = 472), and the distribution of samples subset for Illumina MiSeq DNA sequencing (n = 48).

| Hive | Northings | Eastings | DGGE sample count | Illumina sample count |
| --- | --- | --- | --- | --- |
| AW001 | 346 | 525 | 18 | 3 |
| CM001 | 368 | 513 | 12 | 2 |
| DR001 | 438 | 518 | 21 | 1 |
| DRY01 | 307 | 855 | 12 | 2 |
| DRY02 | 48 | 926 | 6 | 0 |
| FR001 | 514 | 485 | 24 | 1 |
| GC001 | 272 | 362 | 18 | 1 |
| GH001 | 709 | 505 | 24 | 1 |
| GT001 | 288 | 425 | 28 | 4 |
| HC001 | 594 | 492 | 6 | 0 |
| HC003 | 594 | 492 | 6 | 0 |
| HJ001 | 662 | 468 | 30 | 6 |
| JAH01 | 210 | 625 | 12 | 2 |
| JB001 | 361 | 547 | 18 | 1 |
| JH001 | 210 | 835 | 12 | 7 |
| JM001 | 672 | 550 | 18 | 0 |
| JM002 | 672 | 550 | 1 | 4 |
| JP001 | 889 | 486 | 20 | 2 |
| LW001 | 574 | 438 | 24 | 2 |
| PC001 | 614 | 468 | 30 | 3 |
| PD001 | 533 | 519 | 6 | 0 |
| PM001 | 638 | 474 | 21 | 1 |
| PM002 | 605 | 487 | 24 | 2 |
| PS001 | 783 | 452 | 15 | 1 |
| RL001 | 574 | 438 | 9 | 0 |
| RS001 | 665 | 472 | 12 | 0 |
| YC001 | 753 | 504 | 30 | 1 |
| YC002 | 753 | 504 | 6 | 0 |
| YC003 | 753 | 504 | 9 | 0 |
