# Supplementary material for: Bacterial communities associated with honeybee food stores are correlated with land use
Source: Ecol Evol. 2018 Apr 16;8(10):4743–56. doi: 10.1002/ece3.3999 (PMC5980251; doi:10.1002/ece3.3999)
Supplement: Supplementary file 6 [file ECE3-8-4743-s006.doc]

Table S2. Identification of bands excised from DGGE gels, as identified by partial 16S rRNA gene sequencing. Closest homologue identity for each band was assigned, identity scores below 80 were assigned “Uncultured bacterium” as their identity.

| OTU | DGGE Gel Position | Closest 16S Homologue on GenBank | Identity score | Frequency in present population (%; n=472) | Accession number |
| --- | --- | --- | --- | --- | --- |
| BB1 | a1 | Uncultured bacterium clone BIGH1473 | 95 | 19.07 | KF881801 |
| BB2 | a2 | Uncultured bacterium | 86 | 4.45 | - |
| BB3 | a3 , a4 | Uncultured bacterium clone FL5Ad11_3665 | 97, 98 | 11.86 | KF881802 |
| BB4 | a5 , a6 , a7 | Uncultured bacterium clone BIGH1473 | 96, 98, 97 | 7.42 | KF881803 |
| BB5 | a8 | *Enterobacter* sp. p96_C03 | 99 | 2.54 | KF881804 |
| BB6 | a9 | Uncultured bacterium clone Phil_e14 | 93 | 3.18 | KF881805 |
| BB7 | a10 | *Lactobacillus* sp. Fhon13N | 99 | 1.69 | KF881806 |
| BB8 | b1 | Uncultured bacterium | 82 | 1.69 | KF881807 |
| BB9 | b2 | Uncultured bacterium clone BIGH1473 | 95 | 1.48 | KF881808 |
| BB10 | b3 | Uncultured proteobacterium clone DDOUFD08 | 98 | 1.48 | KF881809 |
| BB11 | b4 | Uncultured bacterium clone Ontario1287 | 99 | 3.60 | KF881810 |
| BB12 | b5 , b7 | *Lactobacillus* sp. YH-15 | 98, 98 | 14.62 | KF881811 |
| BB13 | b6 | Uncultured bacterium | 84 | 16.53 | KF881812 |
| BB14 | b8 | Uncultured bacterium clone Ontario1283 | 99 | 5.08 | KF881813 |
| BB15 | b9 | *Clostridium* sp. SL29 | 88 | 3.39 | KF881814 |
| BB16 | b10 , b11 | *Lactobacillus* sp. 80-30 | 99, 98 | 32.63 | KF881815 |
| BB17 | c1 , c2 | Uncultured bacterium | 85 | 5.72 | KF881816 |
| BB18 | c3 | Uncultured bacterium | 87 | 8.69 | KF881817 |
| BB19 | c4 | *Pseudomonas* sp. MFS3 | 99 | 6.78 | KF881818 |
| BB20 | c5 | *Acinetobacter* sp. HYN18 | 99 | 4.66 | KF881819 |
| BB21 | c6 | *Acinetobacter* sp. SAP 971.1 | 97 | 3.18 | KF881820 |
| BB22 | c7 | *Pseudomonas* sp. MFS3 | 99 | 2.54 | KF881821 |
| BB23 | c8 | Uncultured bacterium clone FL5Ad11_3665 | 99 | 16.31 | KF881822 |
| BB24 | d1 , d2 , d3 | *Frischella* sp. PEB0191 | 99, 99, 99 | 20.76 | KF881823 |
| BB25 | d4 | Uncultured bacterium | 89 | 8.90 | KF881824 |
| BB26 | d5 , d6 | Uncultured bacterium | 76, 81 | 4.66 | - |
| BB27 | d7 | Uncultured bacterium clone FL5Ad11_3665 | 98 | 3.39 | KF881825 |
| BB28 | d8 | *Pseudomonas* sp. OF38 | 97 | 1.27 | KF881826 |
| BB29 | d9 | Uncultured bacterium | 88 | 35.17 | KF881827 |
| BB30 | e1 | *Pseudomonas* sp. cc 1451 | 92 | 6.36 | KF881828 |
| BB31 | e2 | Uncultured bacterium | 81 | 2.54 | KF881829 |
| BB32 | e3 | Uncultured bacterium | 67 | 3.81 | - |
| BB33 | e4 , e5 | Uncultured bacterium clone Ontario1287 | 99, 98 | 26.06 | KF881830 |
| BB34 | f1 | *Arsenophonus* sp. ATCC 49151 | 94 | 21.19 | KF881831 |
| BB35 | f2 | *Massilia* sp. FP2-21-4 | 96 | 22.67 | KF881832 |
| BB36 | f3 | Uncultured bacterium | 80 | 17.58 | - |
| BB37 | f4 | Uncultured bacterium | 80 | 22.46 | KF881833 |
| BB38 | f5 | *Enterobacter* sp. p96_C03 | 99 | 18.22 | KF881834 |
| BB39 | f6 | Uncultured bacterium clone BIGH1473 | 99 | 5.30 | KF881835 |
| BB40 | f7 | *Erwinia*  sp. LMG 2688 | 97 | 12.29 | KF881836 |
| BB41 | f8 | Uncultured bacterium | 70 | 18.64 | - |
| BB42 | g1 , g2 , g3, g4 , g5 , g6, g7 | Uncultured bacterium clone BIGH1473 | 99, 99, 93, 99, 97, 98, 92 | 58.05 | KF881837 |
| BB43 | g8 , g9 , g10 , g11 | Uncultured proteobacterium clone GASP-MB1W1_C05 | 97, 97, 97, 98 | 39.62 | KF881838 |
| BB44 | h1 | Uncultured bacterium | 85 | 9.75 | KF881839 |
| BB45 | h2 | *Rosenbergiella* sp. strain CdVSA | 99 | 18.22 | KF881840 |
| BB46 | h3 | *Gilliamella* sp. Strain wkB1 | 99 | 6.22 | KF881841 |
| BB47 | h4 | Uncultured gamma proteobacterium clone gMD365.2_c22 | 99 | 9.96 | KF881842 |
| BB48 | h5 | *Erwinia* sp. CF03 | 99 | 3.18 | KF881843 |
| BB49 | h6, h7 | *Enterobacter* sp. p95_C06 | 98, 98 | 7.63 | KF881844 |
| BB50 | h8 | Uncultured bacterium clone FL5Ad11_3665 | 99 | 8.47 | KF881845 |
| BB51 | h9 | *Enterobacter* sp. p62_B05 | 98 | 4.24 | KF881846 |
| BB52 | h10 | Uncultured bacterium | 87 | 5.72 | KF881847 |
| BB53 | h11 | Uncultured bacterium clone FL5Ad11_3665 | 98 | 2.97 | KF881848 |
