# Supplementary material for: Bacterial communities associated with honeybee food stores are correlated with land use
Source: Ecol Evol. 2018 Apr 16;8(10):4743–56. doi: 10.1002/ece3.3999 (PMC5980251; doi:10.1002/ece3.3999)
Supplement: Supplementary file 7 [file ECE3-8-4743-s007.docx]

Table S3. Full PCA factor loadings for landscape cover analysis.

| **500m** | Comp.1 | Comp.2 | Comp.3 | Comp.4 | Comp.5 | Comp.6 | Comp.7 | Comp.8 |  |  |  |  |  |
| --- | --- | --- | --- | --- | --- | --- | --- | --- | --- | --- | --- | --- | --- |
| Acid grassland | -0.071 | 0.084 | -0.695 | -0.332 | -0.285 | -0.491 | 0.260 | -0.060 |  |  |  |  |  |
| Broad leaf woodland | -0.161 | -0.479 | -0.402 | 0.172 | -0.234 | 0.609 | 0.068 | -0.352 |  |  |  |  |  |
| Fresh water | -0.072 | -0.537 | 0.006 | 0.506 | -0.048 | -0.578 | -0.337 | -0.028 |  |  |  |  |  |
| Improved grassland | -0.588 | 0.179 | 0.107 | -0.217 | 0.274 | -0.115 | -0.272 | -0.634 |  |  |  |  |  |
| Littoral sand | 0.439 | -0.211 | 0.218 | -0.526 | -0.427 | -0.011 | -0.445 | -0.242 |  |  |  |  |  |
| Neutral grassland | -0.269 | 0.341 | 0.326 | 0.304 | -0.761 | -0.036 | 0.154 | -0.091 |  |  |  |  |  |
| Rough grassland | -0.127 | -0.492 | 0.435 | -0.284 | 0.049 | -0.157 | 0.661 | -0.084 |  |  |  |  |  |
| Urban | 0.580 | 0.201 | -0.006 | 0.327 | 0.152 | -0.122 | 0.286 | -0.629 |  |  |  |  |  |

| **3000m** | Comp.1 | Comp.2 | Comp.3 | Comp.4 | Comp.5 | Comp.6 | Comp.7 | Comp.8 | Comp.9 | Comp.10 | Comp.11 | Comp.12 | Comp.13 |
| --- | --- | --- | --- | --- | --- | --- | --- | --- | --- | --- | --- | --- | --- |
| Acid grassland | 0.26 | 0.28 | -0.29 | -0.05 | -0.20 | -0.24 | -0.39 | 0.70 | 0.11 | 0.00 | 0.11 | 0.00 | 0.00 |
| Arable horticultural farmland | 0.19 | 0.11 | 0.53 | 0.13 | 0.26 | -0.25 | -0.04 | 0.08 | -0.06 | -0.06 | 0.10 | 0.18 | -0.68 |
| Broad leaf woodland | 0.34 | -0.04 | 0.19 | -0.59 | -0.18 | -0.18 | 0.32 | 0.02 | 0.31 | 0.04 | -0.48 | 0.00 | 0.00 |
| Urban | 0.29 | 0.37 | -0.17 | 0.11 | 0.32 | 0.30 | 0.19 | -0.03 | 0.10 | 0.03 | -0.07 | 0.68 | 0.18 |
| Coniferous woodland | 0.37 | 0.01 | -0.08 | -0.52 | 0.09 | 0.12 | -0.38 | -0.30 | -0.54 | -0.11 | 0.17 | 0.00 | 0.00 |
| Dry scrub heath | 0.29 | 0.37 | -0.17 | 0.11 | 0.32 | 0.30 | 0.19 | -0.03 | 0.10 | 0.03 | -0.07 | -0.68 | -0.18 |
| Fresh water | 0.19 | 0.11 | 0.53 | 0.13 | 0.26 | -0.25 | -0.04 | 0.08 | -0.06 | -0.06 | 0.10 | -0.18 | 0.68 |
| Improved grassland | 0.12 | -0.49 | 0.02 | 0.16 | 0.34 | 0.19 | -0.40 | 0.19 | -0.04 | 0.29 | -0.53 | 0.00 | 0.00 |
| Litoral rock | -0.34 | 0.25 | 0.06 | -0.28 | 0.27 | -0.03 | -0.50 | -0.27 | 0.58 | 0.07 | 0.06 | 0.00 | 0.00 |
| Litoral sand | -0.34 | 0.25 | 0.20 | -0.31 | 0.06 | 0.19 | 0.16 | 0.30 | -0.31 | 0.65 | 0.08 | 0.00 | 0.00 |
| Neutral grassland | -0.07 | -0.44 | -0.14 | -0.32 | 0.50 | 0.05 | 0.27 | 0.34 | 0.17 | -0.23 | 0.40 | 0.00 | 0.00 |
| Rough grassland | 0.18 | -0.12 | 0.41 | 0.01 | -0.37 | 0.69 | -0.13 | 0.13 | 0.26 | -0.08 | 0.25 | 0.00 | 0.00 |
| Semi-litoral sands | -0.39 | 0.24 | 0.12 | -0.12 | 0.07 | 0.22 | -0.05 | 0.26 | -0.23 | -0.63 | -0.43 | 0.00 | 0.00 |

| **10000m** | Comp.1 | Comp.2 | Comp.3 | Comp.4 | Comp.5 | Comp.6 | Comp.7 | Comp.8 | Comp.9 | Comp.10 | Comp.11 | Comp.12 | Comp.13 |
| --- | --- | --- | --- | --- | --- | --- | --- | --- | --- | --- | --- | --- | --- |
| Acid grassland | -0.380 | -0.234 | 0.144 | 0.106 | -0.316 | 0.115 | 0.308 | 0.097 | -0.101 | 0.279 | 0.063 | -0.672 | -0.085 |
| Arable horticultural farmland | 0.352 | -0.150 | 0.278 | 0.185 | 0.089 | -0.507 | -0.289 | 0.051 | -0.338 | 0.173 | 0.204 | -0.115 | -0.438 |
| Broad leaf woodland | -0.356 | 0.309 | -0.113 | -0.085 | 0.197 | -0.343 | 0.076 | 0.128 | 0.128 | -0.149 | 0.725 | -0.068 | 0.101 |
| Urban | 0.041 | -0.406 | 0.018 | 0.034 | 0.671 | 0.223 | 0.049 | -0.148 | 0.375 | -0.196 | 0.049 | -0.251 | -0.251 |
| Coniferous woodland | -0.313 | 0.379 | 0.051 | 0.046 | 0.129 | -0.365 | 0.255 | -0.461 | 0.147 | 0.167 | -0.395 | 0.045 | -0.346 |
| Dry scrub heath | -0.300 | -0.280 | 0.258 | -0.029 | -0.387 | -0.009 | -0.315 | -0.506 | 0.021 | -0.467 | 0.152 | 0.108 | -0.071 |
| Fresh water | -0.274 | -0.150 | -0.223 | -0.526 | 0.321 | 0.032 | -0.096 | -0.149 | -0.653 | 0.069 | -0.081 | -0.021 | 0.041 |
| Improved grassland | 0.074 | 0.427 | 0.216 | -0.390 | -0.108 | 0.445 | -0.035 | 0.174 | -0.018 | -0.130 | 0.108 | -0.037 | -0.585 |
| Litoral rock | -0.046 | 0.333 | 0.038 | 0.647 | 0.209 | 0.349 | 0.024 | -0.179 | -0.459 | -0.211 | 0.078 | -0.054 | 0.071 |
| Litoral sand | 0.162 | 0.035 | -0.577 | 0.028 | -0.189 | -0.241 | 0.060 | 0.104 | -0.073 | -0.562 | -0.218 | -0.351 | -0.198 |
| Neutral grassland | 0.312 | 0.333 | 0.229 | -0.245 | 0.056 | -0.053 | -0.263 | -0.309 | 0.099 | -0.004 | -0.070 | -0.560 | 0.425 |
| Rough grassland | -0.421 | 0.107 | -0.134 | 0.179 | 0.083 | 0.041 | -0.733 | 0.306 | 0.177 | 0.127 | -0.239 | -0.112 | -0.064 |
| Semi-litoral sands | 0.178 | 0.017 | -0.564 | 0.058 | -0.179 | 0.215 | -0.165 | -0.444 | 0.109 | 0.433 | 0.337 | -0.021 | -0.174 |
