# Supplementary material for: Bacterial communities associated with honeybee food stores are correlated with land use
Source: Ecol Evol. 2018 Apr 16;8(10):4743–56. doi: 10.1002/ece3.3999 (PMC5980251; doi:10.1002/ece3.3999)
Supplement: Supplementary file 8 [file ECE3-8-4743-s008.docx]

Table S4. Proportion of sequence reads (MiSeq) and bands (DGGE) assigned to the 20 most common genera detected by each of these sequencing technologies.

|  |  |  |  |  |
| --- | --- | --- | --- | --- |
| Genus | DGGE Bands | DGGE % | MiSeq Read Count | MiSeq % |
| *Acinetobacter* | 156 | 13.36 | 76114 | 1.67 |
| *Arsenophonus* | 121 | 10.36 | 368826 | 8.09 |
| *Bacillus* |  |  | 3591 | 0.08 |
| *Clostridium* | 16 | 1.37 | 34280 | 0.75 |
| *Enterobacter* | 154 | 13.18 | 2197 | 0.05 |
| *Erwinia* | 73 | 6.25 | 119927 | 2.63 |
| *Escherichia* |  |  | 36375 | 0.75 |
| *Frischella* | 126 | 10.77 | 255821 | 5.61 |
| *Lactobacillus* | 231 | 19.77 | 122784 | 2.69 |
| *Massilia* | 107 | 9.16 | 23159 | 0.51 |
| *Phyllobacterium* |  |  | 20412 | 0.45 |
| *Pseudomonas* | 155 | 13.26 | 409173 | 8.97 |
| *Raoultella* |  |  | 52032 | 1.14 |
| *Rosenbergiella* | 29 | 2.51 | 485 | 0.01 |
| *Saccharibacter* |  |  | 53030 | 1.16 |
| *Serratia* |  |  | 18726 | 0.41 |
| *Sphingomonas* |  |  | 13417 | 0.29 |
| *Staphylococcus* |  |  | 4044 | 0.09 |
| *Streptococcus* |  |  | 5276 | 0.12 |
| *Tatumella* |  |  | 24994 | 0.55 |
| Non-DGGE |  |  | 4559864 | 8.09 |
