# Supplementary material for: Bacterial communities associated with honeybee food stores are correlated with land use
Source: Ecol Evol. 2018 Apr 16;8(10):4743–56. doi: 10.1002/ece3.3999 (PMC5980251; doi:10.1002/ece3.3999)
Supplement: Supplementary file 9 [file ECE3-8-4743-s009.docx]

**Supplementary Materials 1: Landscape analysis methods**

To estimate the correlation between land cover and DGGE-estimated bacterial community composition, we used data sourced from the Countryside Survey Land Cover Map 2007 (Morton et al. [2011](http://onlinelibrary.wiley.com/doi/10.1002/ece3.1293/full#ece31293-bib-0043)). Improved grasslands, woodland, and urban environments primarily dominate the areas surrounding hives within the study region.

We performed analyses based on the composition and configuration of different land cover classes within three radial buffer zones (defined as circular areas comprising the landscape surrounding each hive in the study). The primary buffer zone for analysis was 3 km in radius. Honeybee foraging (and therefore interaction with the environment) is most efficient at 3 km (Steffan-Dewenter et al. [2002](http://onlinelibrary.wiley.com/doi/10.1002/ece3.1293/full#ece31293-bib-0065)), but they are capable of foraging up to 10 km from the hive (Seeley [1986](http://onlinelibrary.wiley.com/doi/10.1002/ece3.1293/full#ece31293-bib-0060)). Only 10% of the foraging activity occurs within 0.5 km of the hive, 50% forage at >6 km, 25% more than 7.5 km, and 10% more than 9.5 km from the hive (Beekman and Ratnieks [2000](http://onlinelibrary.wiley.com/doi/10.1002/ece3.1293/full#ece31293-bib-0007)). To test for potential localized effects around each hive, we included an inner buffer zone of 0.5 km and a 10-km buffer zone to test beyond the scale of the study described by Steffan-Dewenter et al. ([2002](http://onlinelibrary.wiley.com/doi/10.1002/ece3.1293/full#ece31293-bib-0065)). Land cover classes that accounted for <0.5% of total cover within a buffer zone were excluded from analysis. Therefore, at 0.5 km eight classes were included, at 3 km 13 classes, and at 10 km 13 classes. These classes were assigned a three letter code:

| AGL | Acid grassland | |
| --- | --- | --- |
| ARH | Arable horticultural farmland | |
| BLW | Broad leaf woodland | |
| BUG | Urban |  |
| CNW | Coniferous woodland | |
| DSH | Dry scrub heath | |
| FWT | Fresh water | |
| IGL | Improved grassland | |
| LTR | Litoral rock | |
| LTS | Litoral sand | |
| NGL | Neutral grassland | |
| RGL | Rough grassland | |
| SLS | Semi-litoral sands | |

To quantify how community composition varied geographically, we used the Countryside Survey 2007 Land Cover Map to describe local landscape composition (Morton et al. 2011). Countryside Survey data ascribe total land cover (km²) to different landscape types (Donkersley et al., 2014; Morton et al. 2011). Buffer zones with radii of 500 m, 3 km, and 10 km around each hive had values for total land cover in raw area (km²) converted to relative land cover (%) and arcsine transformed to normalize the residuals for statistical analysis.

The landscape composition variables were analysed in a principal components analysis (PCA). PCA produced 13 components, the first six of which explained 82% of the landscape cover variance (Table 2). The full loadings for all the principal components are available below in Table S1M. The first six components were included in as explanatory variables in linear models tested against the microbial community composition as response variables.

**References**

Beekman, M., and F. L. W. Ratnieks. 2000. Long-range foraging by the honey-bee, *Apis mellifera* L. *Funct. Ecol.* **14**:490–496.

Donkersley, P., G. Rhodes, R. W. Pickup, K. C. Jones, and K. Wilson. 2014. Honeybee nutrition is linked to landscape composition. Ecology and Evolution **4**:4195-4206.

Morton, R. D., C. Rowland, C. Wood, L. Meek, C. Marston, G. Smith, et al. 2011. CS Technical Report No 11/07: Final Report for LCM2007 – the new UK land cover map. NERC/Centre for Ecology & Hydrology (CEH Project Number NEC03259).

Seeley, T. D. 1986. Social foraging by honeybees – how colonies allocate foragers among patches of flowers. *Behav. Ecol. Sociobiol.* **19**:343–354.

Steffan-Dewenter, I., U. Münzenberg, C. Bürger, C. Thies, and T. Tscharntke. 2002. Scale-dependent effects of landscape context on three pollinator guilds. *Ecology* **83**:1421–1432.
